# Histological evidence for a dynamic dental battery in hadrosaurid dinosaurs

**DOI:** 10.1038/s41598-017-16056-3

**Published:** 2017-11-17

**Authors:** Katherine Bramble, Aaron R. H. LeBlanc, Denis O. Lamoureux, Mateusz Wosik, Philip J. Currie

**Affiliations:** 1 Department of Biological Sciences, University of Alberta, Edmonton, Alberta T6G 2E9 Canada; 2 St. Joseph’s College, University of Alberta, Edmonton, Alberta T6G 2J5 Canada; 3 Department of Ecology and Evolutionary Biology, University of Toronto, 100 Queen’s Park, Toronto, ON M5S 2C6 Canada

## Abstract

The first histological study of an entire hadrosaurid dental battery provides a comprehensive look at tooth movement within this complex structure. Previous studies have focused on isolated teeth, or *in-situ* batteries, but this is the first study to examine an entire dental battery of any dinosaur. The absence of direct tooth-to-tooth contact across the entire battery and a unique arrangement of the dental tissues in hadrosaurids led us to compare their teeth with the ever-growing incisors of mammals. The similarity in the distributions of tissues along the incisor, coupled with continuous eruption, make for helpful comparisons to hadrosaurid teeth. The mammalian ever-growing incisor can be used as a model to extrapolate the soft tissue connections and eruptive mechanisms within the hadrosaurid dental battery. Serial sections across the adult dental battery reveal signs of gradual ontogenetic tooth migration. Extensive remodeling of the alveolar septa and the anteroposterior displacement of successive generations of teeth highlight the gradual migration of tooth generations within the battery. These eruptive and ontogenetic tooth movements would not be possible without a ligamentous connection between successive teeth and the jaws, underscoring the dynamic nature of one of the most unique and complex dental systems in vertebrate history.

## Introduction

Hadrosaurid dinosaurs were among the dominant megaherbivores of most Late Cretaceous ecosystems and their specialized dentitions likely attributed to their success^1–3^. Their dentition was arranged in dental batteries, with up to 60 closely spaced vertical columns of diamond shaped teeth per jaw. Each column of teeth – a tooth family, sensu Edmund^4^– was interlocked with the neighboring tooth families and contained three to six successional teeth^1,5^. The crown-root angle of the dentary teeth, with respect to the occlusal surface, ranged from 120° to 140° in hadrosaurines and is greater than 145° in lambeosaurines^1^. This angle permitted multiple teeth of each tooth family to be functional simultaneously^6,7^. Some species, such as *Edmontosaurus annectens*, had up to 60 tooth families with approximately 300 teeth per dentary^1,5^. This complex dental system increased the efficiency of processing tough vegetation, including siliceous phytoliths and the soil attached to low-lying vegetation^8,9^, as well as crustaceans^10^.

The hadrosaurid dental battery was originally considered to be a solid block with the crowns and roots of adjacent teeth being cemented together^2,7,11^. However, this view has been scrutinized more recently^3^. Moreover, several histological studies have established homology of dental tissues and tooth attachment tissues (alveolar bone, cementum, and periodontal ligament) between dinosaurs, mammals, and other archosaurs^2,12–18^. When all of these tissues are present, they provide a flexible connection between the tooth and its socket that is mediated by a periodontal ligament, a feature that LeBlanc *et al*.^3^ extended to the complex dental batteries of hadrosaurids.

The traditional model of a rigid dental battery with teeth cemented together^2,7,11^ fails to explain the addition of tooth families through ontogeny, constant tooth eruption, and the abundance of isolated hadrosaurid teeth from Late Cretaceous vertebrate microsites^3,19–21^. The dental battery must have been a dynamic structure, adding over 30 tooth families and undergoing constant tooth wear and replacement^22^. However, the mechanisms of this movement are not well understood. According to the recent model of LeBlanc *et al*.^3^, periodontal ligaments would connect all of the teeth within the battery and provide an agent through which teeth could continuously erupt towards the grinding surface. Confirming the presence of such a soft tissue connection in hadrosaurids is difficult, however, without an extant analogue to show how such a ligament would connect individual teeth within such an unusual dentition.

To test the traditional and recent models, the first known histological thin sections of an entire adult dental battery, as well as a nearly complete perinatal dental battery, were prepared along the occlusal (transverse) plane. If the dental battery was a rigid structure then cementum, or another hard tissue, should be found between the teeth. However, if the teeth were separated by sediment-filled spaces, this would suggest that periodontal ligaments, which mediate tooth movement, held the dental batteries together^17^. In order to extrapolate the soft tissues of the hadrosaurid tooth, these histological thin sections were then compared with crocodilians for an extant archosaur comparison and lagomorph incisors due to their similarity in the arrangement of attachment tissues that hold the continuously erupting tooth within the jaw.

## Results

**Figure 1:**
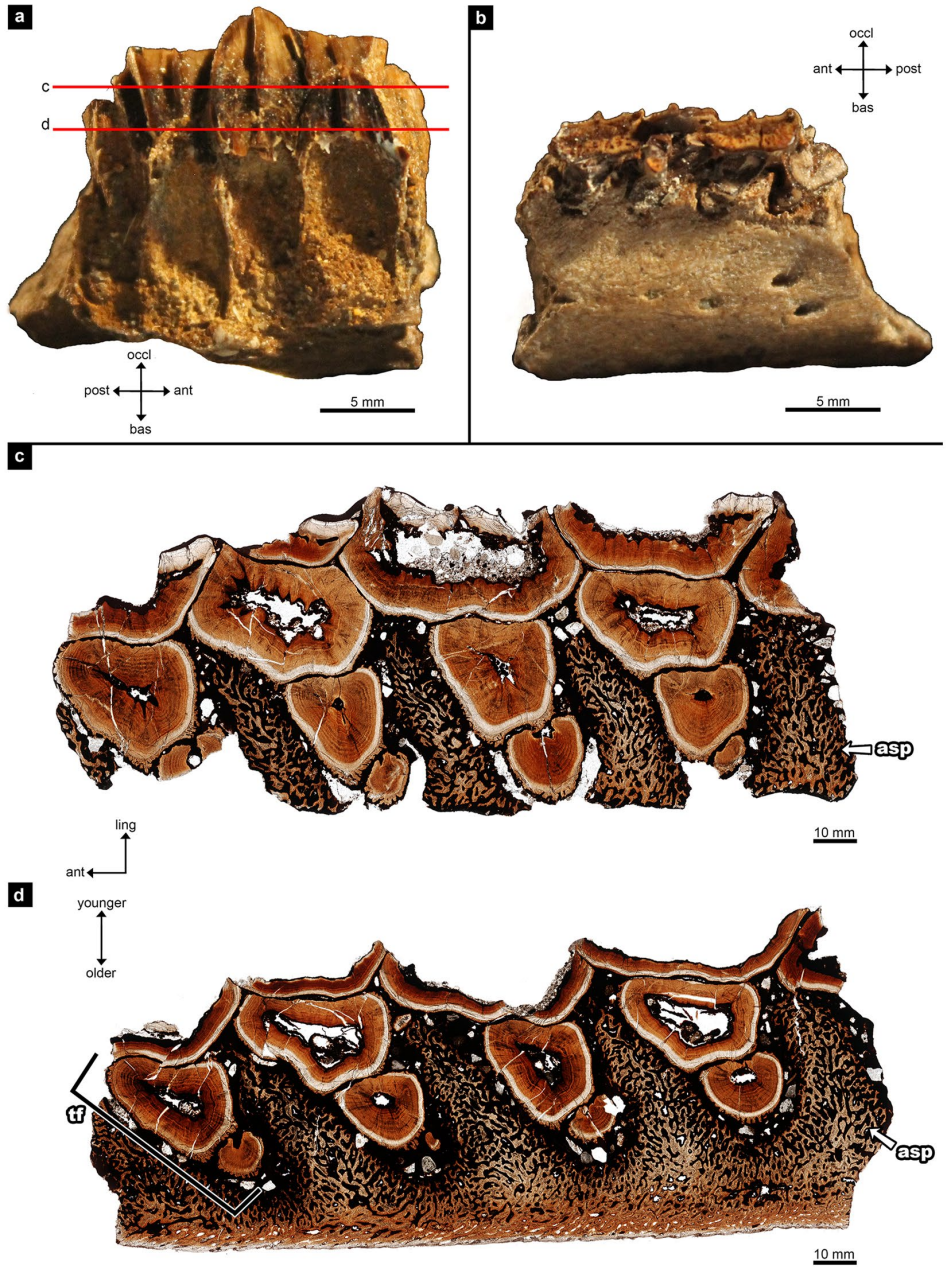
The perinatal hadrosaurid right dentary (UALVP 54419). (**a**) lingual and (**b**) labial views of dentary before histological thin sectioning. Anterior is to the left in (**a**) and to the right in (**b**). The letters c and d in (**a**) correlate to images (**c**) and (**d**). (**c**) occlusal transverse histological thin section; (**d)**, basal transverse histological thin section. ant, anterior; asp, alveolar septum; bas, basal; ling, lingual; occl, occlusal; post, posterior; tf, tooth family. Younger and older refers to the age of the teeth.

**Figure 2:**
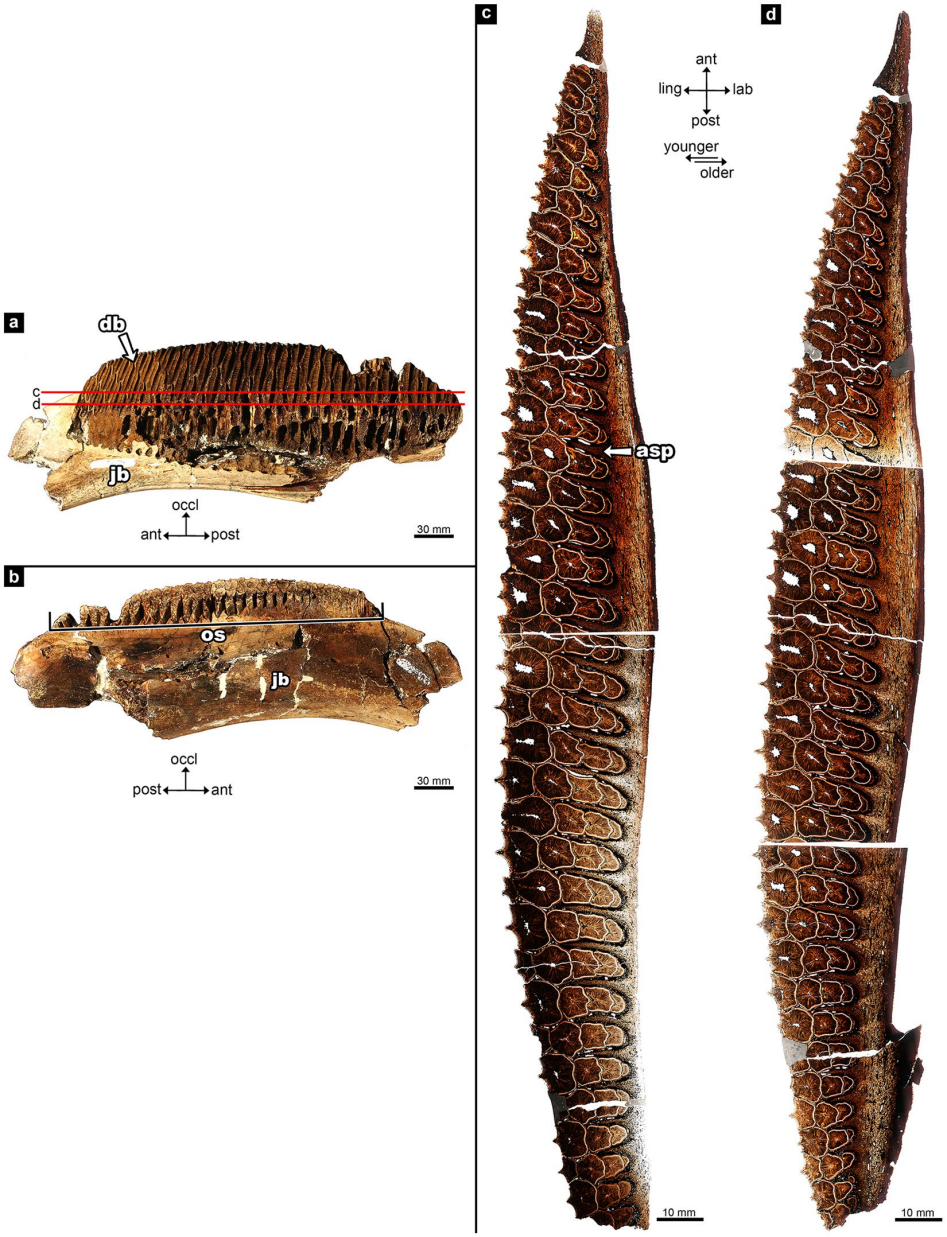
The adult hadrosaurid right dentary (UALVP 56336). (**a**) lingual and (**b**) labial views of dentary before histological thin sectioning. Anterior is to the left in (**a**) and to the right in (**b**). The letters c and d in (**a**) correlate to images (**c**) and (**d**). (**c**) occlusal transverse histological thin section; (**d**) basal transverse histological thin section. ant, anterior; asp, alveolar septum; bas, basal; db, dental battery; jb, jaw bone; lab, labial; ling, lingual; occl, occlusal; os, occlusal surface; post, posterior. Younger and older refers to the age of the teeth.

The perinatal dentary had five tooth families preserved. Only one tooth per family was in occlusion, but three or four teeth were visible within each tooth family in the transverse sections (Fig. 1). The adult dentary had 40 tooth families preserved with two to five teeth per family visible in transverse histological section. The adult dentary had two to four teeth per family in occlusion (Fig. 2). Hadrosaur dental histology has been previously described in detail by Erickson *et al*.^2^, followed by LeBlanc *et al*.^3^, so the focus here is on similarities between the hadrosaurid tooth and lagomorph incisor (*Ochotona sp., Oryctolagus cuniculus*).

### Dental Attachment in the Hadrosaurid Tooth

**Figure 3:**
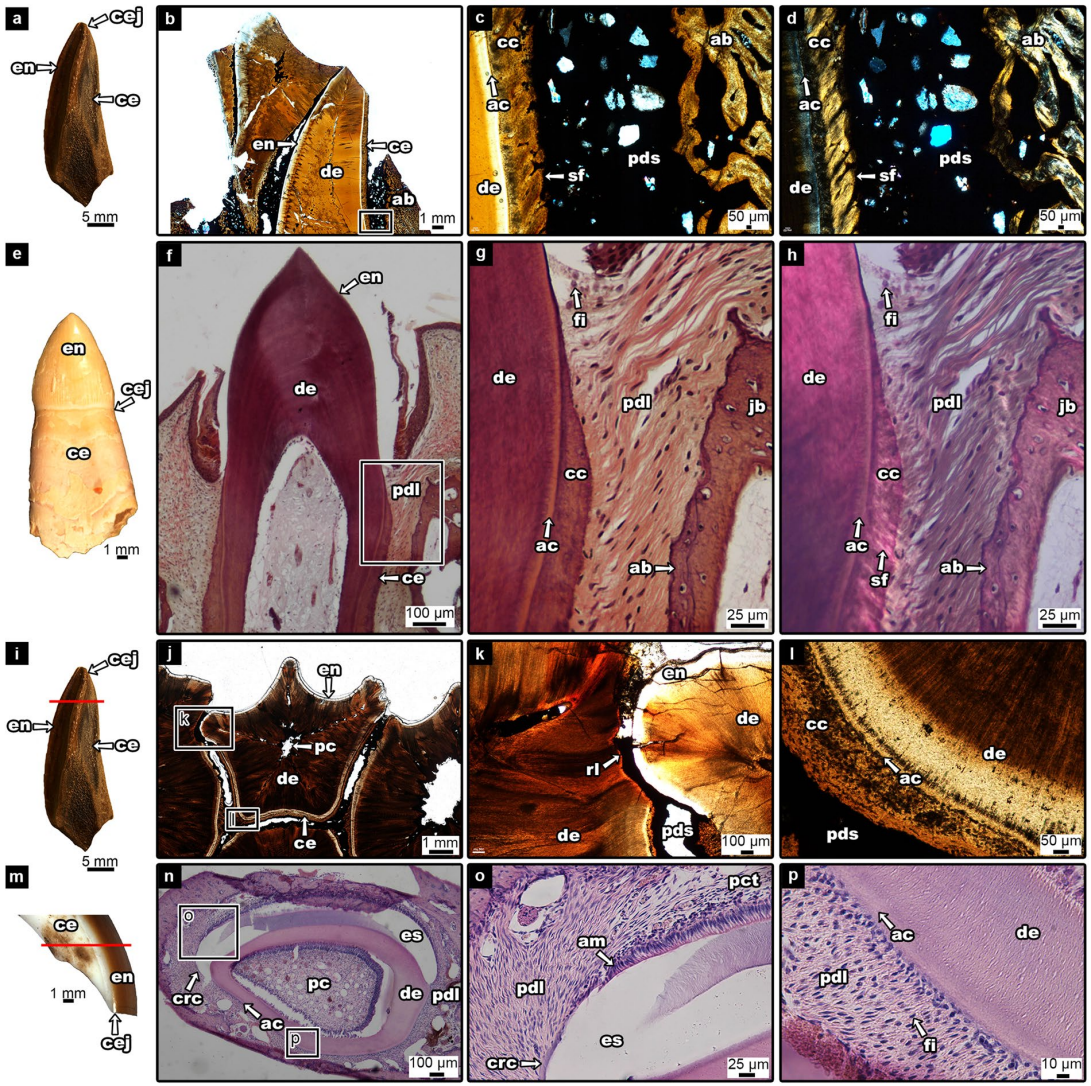
Tooth attachment tissue comparisons between the hadrosaurid dental battery (UALVP 56336), a crocodilian (*Caiman sclerops*), and a lagomorph (*Ochotona sp*.) (**a**) hadrosaurid tooth in coronal view; (**b**) histological thin section of a hadrosaurid maxilla (ROM 00696) in coronal view (image flipped upside down to match dental tissue orientation of the dentary); (**c**) magnified image of box in (**b**); (**d**) magnified image of box in (**b**), cross-polarized; (**e**) alligator tooth; (**f**) histological thin section of crocodilian tooth (*Caiman sclerops*) in coronal view; (**g**) magnified image of box in (**f**); (**h**) magnified image of box in (**f**), cross-polarized; (**i**) hadrosaurid tooth in coronal view; (**j**) histological thin section of hadrosaurid teeth in transverse view; (**k**) magnified image of box-k in (**j**); (**l**) magnified image of box-l in (**j**); (**m**) upper beaver incisor representing a lagomorph incisor, coronal view; (**n**) histological thin section of a lagomorph incisor (*Ochotona sp*.) in transverse view; (**o)** magnified image of box-o in (**n**); (**p**) magnified image of box-p in (**n**). ab, alveolar bone; ac, acellular cementum; am, ameloblasts; cc, cellular cementum; ce, cementum; cej, cemento-enamel junction; crc, coronal cementum; de, dentine; en, enamel; es, enamel space; fi, fibroblast; jb, jaw bone; pc, pulp cavity; pct, periodontal connective tissue; pdl, periodontal ligament; pds, periodontal space; rl, reversal line; sf, Sharpey’s fibers. Red lines in (**i**) and (**m**) indicate direction of succeeding histological thin sections. Note: a beaver tooth was used in (**m**) as the pigmented enamel better demonstrated the cej.

**Figure 4:**
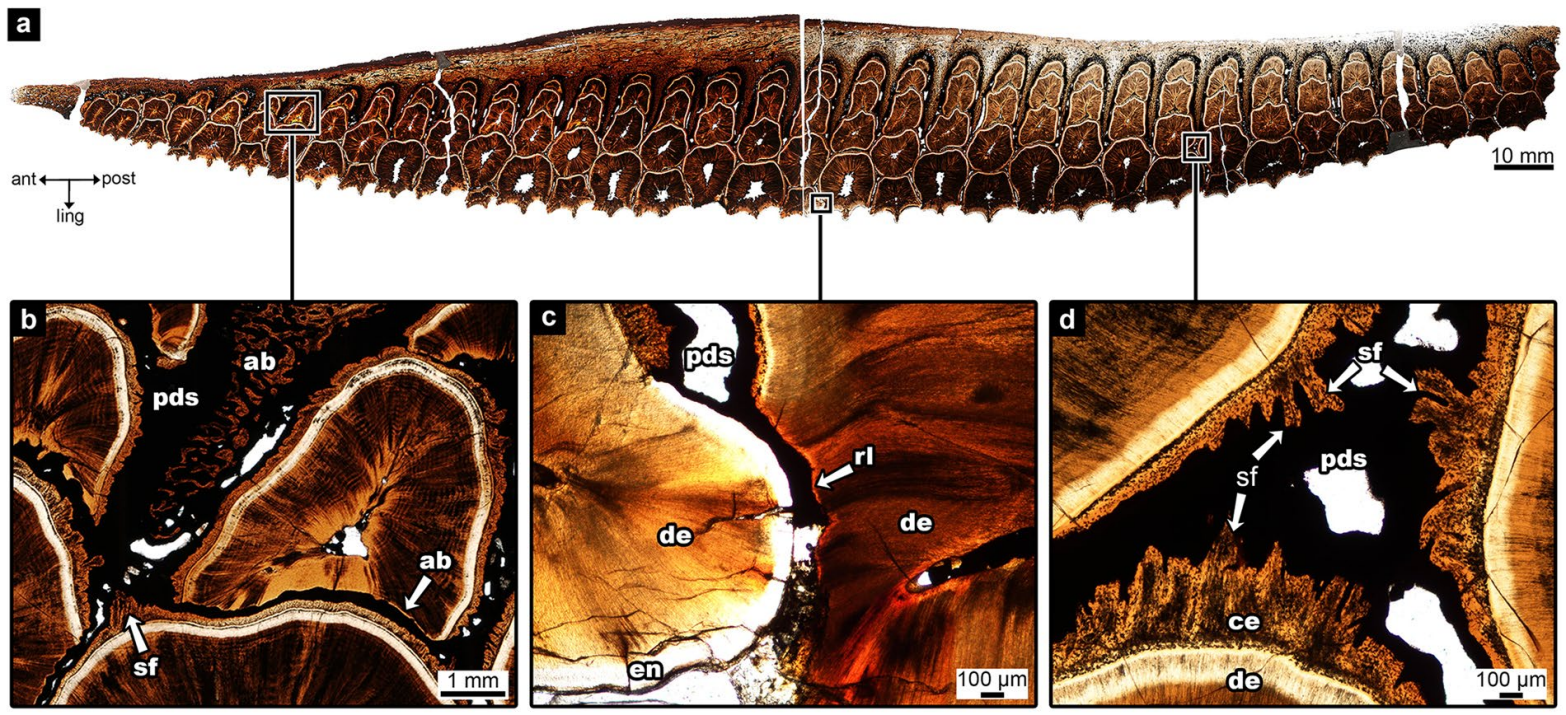
Images of the periodontal space throughout the entire hadrosaurid dental battery (UALVP 56336). (**a**) whole image of the hadrosaurid dental battery, in transverse view; (**b**) magnified image of anterior box in (**a**); (**c**) magnified image of middle box in (**a**); (**b**) magnified image of posterior box in (**a**). ab, alveolar bone; ant, anterior; ce, cementum; de, dentine; en, enamel; ling, lingual; pds, periodontal space; post, posterior; rl, reversal line; sf, Sharpey’s fibers.

Hadrosaurid teeth possess an apomorphic arrangement of enamel and cementum coating each tooth. The teeth have enamel on one side (the lingual surface in dentary teeth, the labial surface in maxillary teeth) and cementum on the other (Fig. 3a). The cementum of the hadrosaurid tooth has two layers – a thin acellular layer and a thick vascularized cellular layer (Fig. 3c,d,l). In hadrosaurids, the alveolar bone forms as wedges between the tooth families called alveolar septa (Fig. 1d). These septa are wedge-shaped, wide at the base, narrow towards the tip, and extend in between tooth families as partitions. In coronal view, partly mineralized collagen fibers, called Sharpey’s fibers, are obliquely angled within the cementum (Fig. 3c,d). A space separates the teeth and alveolar bone and is visible throughout the entire dental battery (Fig. 3c,d,k,l; 4). In areas of the tooth where the cementum has been resorbed by newly developing teeth, a thin layer of alveolar bone is developed for attachment purposes (Fig. 4b).

### Extant Toothed Archosaur

The crocodilian *Caiman sclerops* represents an extant archosaur comparison for tooth attachment tissues with hadrosaurids. Unlike in hadrosaurids, each tooth in *C. sclerops* consists of an enamel-capped crown and a cementum-coated root. Each tooth is suspended within the socket by a network of collagen fiber bundles forming a PDL (Fig. 3e,f). The PDL attachment occurs on all sides of the tooth root. A thin layer of acellular cementum and a thick cellular cementum are visible (Fig. 3g,h); however, the cellular cementum is not vascularized as in hadrosaurids. At one end, the fibers of the PDL are embedded in the cellular cementum, forming Sharpey’s fibers, and the opposite end is embedded in a thin layer of alveolar bone (Fig. 3h). Fibroblasts are visible within the periodontium (Fig. 3g).

### The Lagomorph Ever-Growing Incisor

Similar to hadrosaurid teeth, the “crown” in lagomorph incisors has shifted from the cap-forming position –demonstrated in *C. sclerops* (Fig. 3e) – to one face of the tooth. As such, the cemento-enamel junction (CEJ) is shifted to an apical position (Fig. 3m). The mandibular incisor of a lagomorph features enamel on the labial side and cementum on the lingual side^23,24^ (Fig. 3m). For the hadrosaurid tooth the enamel is on the labial face of the maxillary teeth and the lingual face of the dentary teeth^3,5^ (Fig. 3i). Cementum covers the remaining surface of the tooth.

Histological thin sections of the pika (*Ochotona sp*.) reveal the same attachment tissue arrangement to the hadrosaurid tooth although there is no cellular cementum in the former (Fig. 3n,p). Instead, the cementum consists only of a thin layer of acellular cementum to which the fibers of the PDL attach. As well, the pika and other lagomorphs have thin layers of coronal cementum overlapping a portion of the enamel^23^ (Fig. 3n,o), but coronal cementum is absent in hadrosaurids (Fig. 3j). The PDL of the cementum-covered portion of the tooth is embedded within the acellular cementum and to the surrounding alveolar bone. At the CEJ, the PDL attaches to the coronal cementum that overlaps a small portion of the enamel and anchors into the alveolar bone. However, there is no PDL connection within the enamel-covered portion of the tooth as the ameloblasts prevent any attachment to the enamel (Fig. 3o). Periodontal connective tissues are seen within this space, but they do not form a ligamentous connection (Fig. 3o).

**Figure 5:**
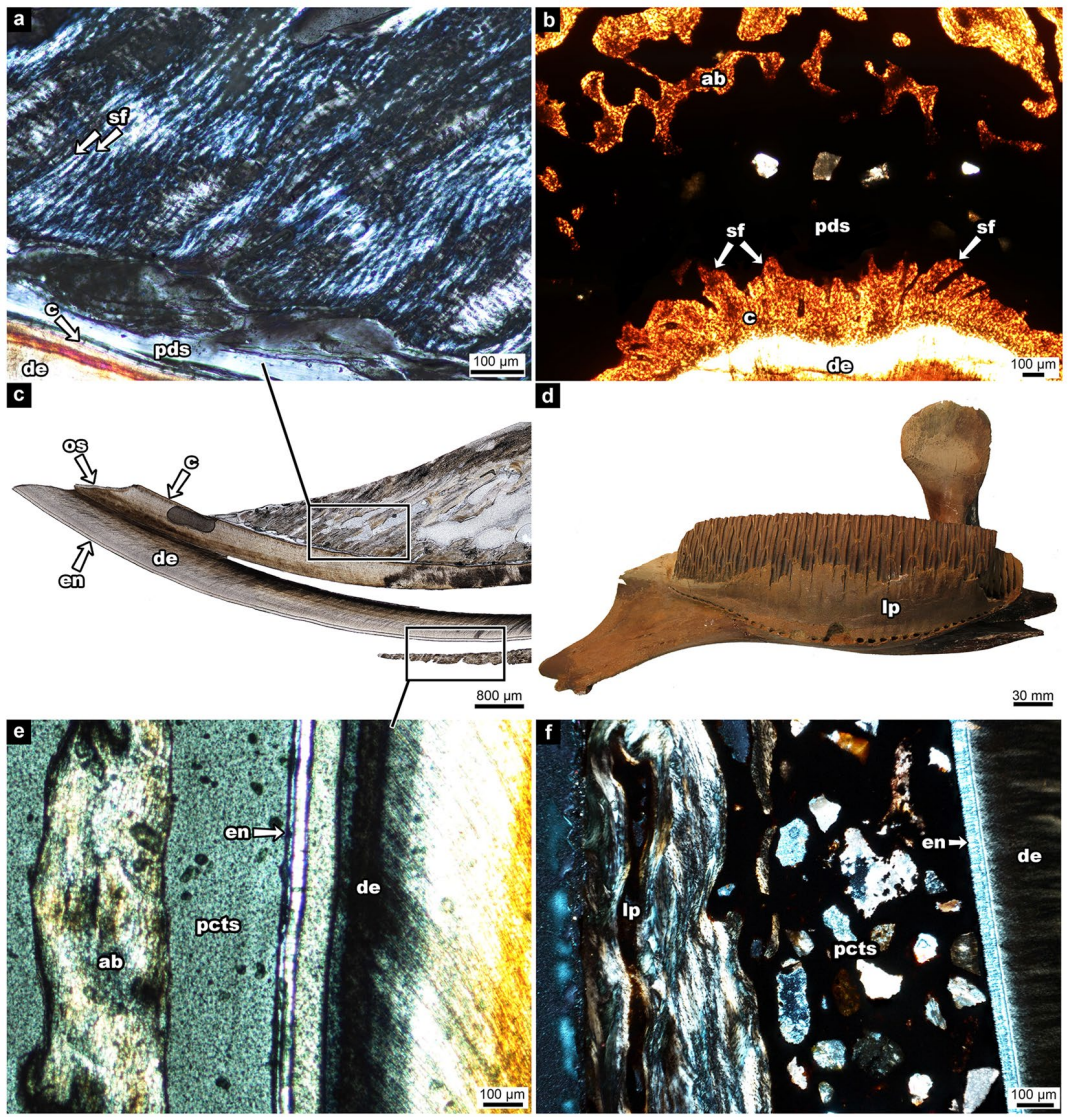
Periodontal ligament attachment in the rabbit (*Oryctolagus cuniculus;* UALVP 56918) and hadrosaurid (UALVP 56336). (**a**) magnified image view of the box in (**c**) of rabbit alveolar bone and the root portion of the incisor; (**b**) corresponding view in the hadrosaurid dental battery between alveolar bone and the tooth root; (**c**) sagittal view of rabbit histological thin section; (**d**) hadrosaurid dental battery (UALVP 11734) showing location of lingual plate [note: the lingual plate in this specimen is incomplete and would reach closer to the occlusal surface in life]; (**e**) magnified image of the box in (**c**) of rabbit alveolar bone and the crown portion of the incisor; (**f**) corresponding view in the hadrosaurid dental battery between the lingual plate and the tooth crown (ROM 3500). (**a**), (**c**), and (**e**) are in sagittal view, while (**b**) is in transverse view, (**d**) is in medial view, and (**f**) is in coronal view. ab, alveolar bone; c, cementum; de, dentine; en, enamel; lp, lingual plate; pcts, periodontal connective tissue space; pds, periodontal space; os, occlusal surface; sf, Sharpey’s fibers.

The PDL is not preserved in the skeletonized rabbit. Sharpey’s fibers are seen in great numbers on the lingual side of the rabbit incisor where the cementum covers the incisor (Fig. 5a), whereas there are no Sharpey’s fibers seen between the enamel-covered labial side of the tooth and the adjacent bone (Fig. 5e). This has also been shown in rodent incisors^24,25^. The space between the enamel of the mammalian ever-growing incisor and the adjacent bone is referred to as the periodontal connective tissue space^24^. This condition is similar in the hadrosaurid dentary, where Sharpey’s fibers were present in the cementum of the teeth and oriented towards the alveolar bone (Fig. 4b) or towards the cementum of other teeth (Figs 4b; 5b). However, Sharpey’s fibers were entirely absent within the thin enamel-facing lingual plate of the dentary (Fig. 5f).

### Tooth Migration Within the Hadrosaurid Dental Battery

**Figure 6:**
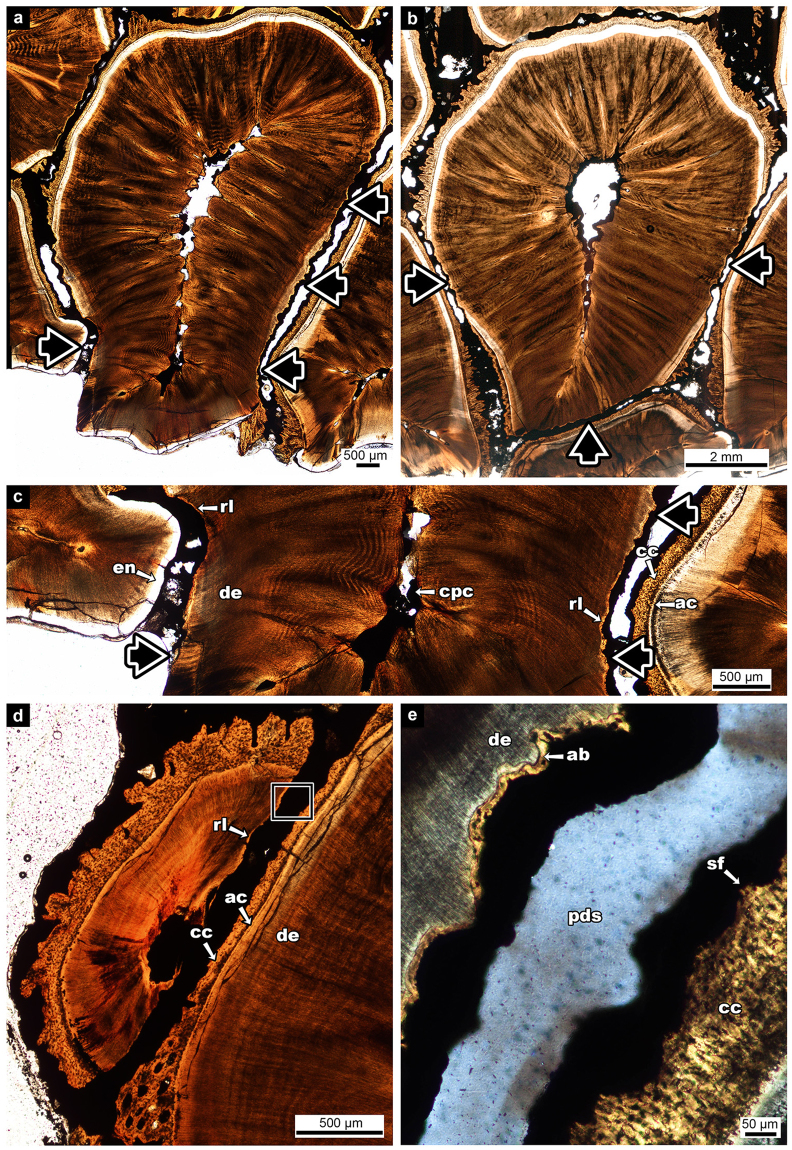
Tooth resorption in the hadrosaurid dental battery (UALVP 56336), all in transverse view. (**a**) an asymmetrically resorbed tooth; (**b**) a symmetrically resorbed tooth; (**c**) magnified view of asymmetrically resorbed tooth; (**d**) half resorbed tooth; (**e**) magnified image of the box in (**d**). Black arrows indicate direction of resorption. ab/rc, alveolar bone or repair cementum; ac, acellular cementum; cc, cellular cementum; cpc, collapsed pulp cavity; de, dentine; en, enamel; pds, periodontal space; rl, reversal line; sf, Sharpey’s fiber.

Transverse sections through entire hadrosaurid dental batteries revealed signs of extensive anteroposterior tooth migration through ontogeny. Anterior teeth of the adult dental battery are asymmetrically resorbed by neighbouring teeth (Fig. 6a). Typically, resorption of older teeth by adjacent younger generations is roughly symmetrical (Fig. 6b) and happens as new teeth are developing on both sides of the older tooth in replacement waves^3^. Tooth resorption is further indicated by the presence of Howship’s lacunae along the dentine surfaces of partially resorbed teeth, as well as a reversal line between the dentine of the partially resorbed tooth and the new layer of alveolar bone that forms subsequent to resorption by adjacent, younger teeth (Fig. 6c). As well, the most anterior tooth in the dental battery is half resorbed by the anteriorly migrating teeth of the second tooth family (Fig. 6d). This partially resorbed tooth still retains a connection to the adjacent tooth via the PDL as evidenced by the uniform spacing and signs of Sharpey’s fibers in cross-polarized light (Fig. 6e). The adjacent tooth also shows signs of resorption anteriorly.

**Figure 7:**
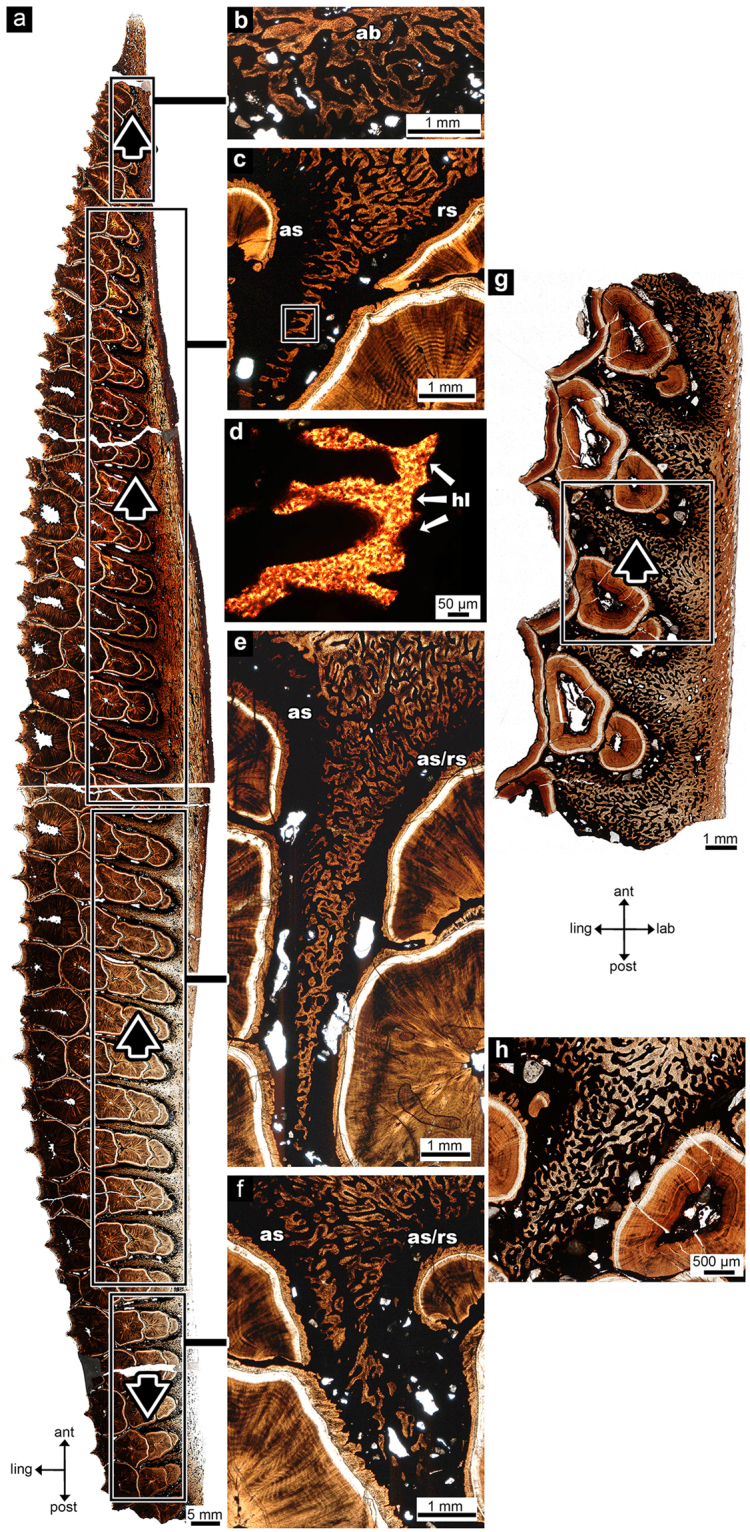
Zones of alveolar bone within the adult (UALVP 56336) and perinatal (54419) hadrosaurid dental batteries, all in transverse view. (**a**) whole section of the adult dental battery; (**b**) magnified image of Zone 1; (**c**) magnified image of Zone 2; (**d**) magnified image of the box in (**c**); (**e**) magnified image of Zone 3; (**f**) magnified image of Zone 4; (**g**) whole section of the perinatal dental battery; (**h**) magnified view of the box in (**g**). Black arrows indicate direction of net movement of alveolar septum. ant, anterior; as, apposition side; hl, Howship’s lacunae; ling, lingual; post, posterior; rs, resorbing side. (**b**), (**c**), (**d**), (**e**), (**f**), and (**h**) anterior is to the left, lingual is down.

The tooth families and alveolar septa also show signs of anteroposterior migration within the hadrosaurid dental battery in transverse section, a feature that has not been documented previously. Rather than forming straight partitions between the teeth, these septa are angled differently in four zones along the dentary (Fig. 7a) and correspond to shifts in the anteroposterior positions of successive tooth generations in the associated tooth families.

Zone 1 consists of the anterior four alveolar septa. These septa do not form complete partitions as no alveolar bone extends between the tooth families (Fig. 7b). The average angle of the alveolar septa in this zone (with the exception of the first) is 102° towards the anterior (range: 101°–105°) for the occlusal thin section and 104° towards the anterior (range: 98°–111°) for the basal thin section. These septa are composed mostly of older, thicker alveolar bone. The tooth families in this zone become progressively more anteriorly situated, so much so that the youngest tooth in each of these families lies anterior to the boundary of adjacent alveolar septa (Fig. 7a).

The second zone consists of the subsequent 15 alveolar septa and tooth families of the adult dentary (Fig. 7c). The first four alveolar septa of this zone are highly angular, ranging from 122°–125° for the occlusal thin section and 125°–130° for the basal thin section, whereas the remaining 11 alveolar septa have an average angle of 107° for the occlusal cut and 111° for the more basal thin section. The anterior side of these wedges shows signs of new bone growth as they are lined with thin woven bone whereas the posterior side of each of these wedges has thicker woven bone with signs of resorption by purported Howship’s lacunae (Fig. 7d). The teeth in this region are also progressively anteriorly inclined in younger tooth generations, but not as pronounced as in the first zone (Fig. 7a).

The third zone consists of the next 11 alveolar septa. These septa show much more subtle anterior inclination. The average angle of the alveolar septa in this zone is 99° towards the anterior (range: 91°–105°) for the occlusal thin section and 102° towards the anterior (range: 99°–108°) for the basal transverse section. Thin woven bone is seen on both the anterior and posterior sides of the alveolar septa; however, on the posterior side it is located only at the base of the septum. Halfway through this zone, the tips of the alveolar septa are rotated towards the posterior (Fig. 7e). However, overall each alveolar septum is inclined anteriorly and the teeth become slightly more anteriorly situated in younger tooth generations (Fig. 7a).

The fourth zone consists of the remaining visible alveolar septa that angle posteriorly, unlike the previous zones (Fig. 7f). The final few alveolar septa do not fully extend between the tooth families, similar to those of the first few anterior alveolar septa. The average angle of the alveolar septa in this zone is 80° – now angled posteriorly – (range: 68°–88°) for the occlusal thin section and 77° (range: 66°–90°) for the basal thin section. These alveolar septa still have new bone forming on the anterior sides of the septa, as well as on the posterior. The teeth in this final zone become progressively more posteriorly situated (Fig. 7a).

In the perinatal dental battery, all of the alveolar septa are angled anteriorly and all tooth families show a pronounced anterior migration of subsequent tooth generations (Fig. 7g). New bone growth is seen on all sides of the alveolar septa (Fig. 7h), signifying rapid growth in all directions. The alveolar septa of the perinatal dental battery are relatively wider between tooth families than in the adult and consist entirely of a heavily vascularized woven bone matrix. However, the septa become more vascular towards the anterior end of the dental battery. The average angle of all alveolar septa is 111.2° towards the anterior (range: 103°–118°) for the occlusal thin section and 123.6° towards the anterior (range: 112°–131°) for the basal thin section.

## Discussion

No extant amniote analogue exists for the hadrosaurid dental battery, which confounds our understanding of how this complex structure evolved and functioned in the living animal. Erickson *et al*.^2^ used ungulate teeth as an analogue for the wear surface produced along the entire dental battery. Whereas this model has aided in our understanding of how the different tooth tissues in ungulates and hadrosaurids wore down to form the grinding surface, ungulate-hadrosaurid comparisons conflate tooth tissue complexity in extant ungulates with the relative simplicity with which an individual hadrosaurid tooth is constructed^13^. This model assumes that the hadrosaurid battery is a static, rigid structure with teeth that are coalesced together and thus behaves like a single ungulate tooth. A rigid battery composed of coalesced teeth is inconsistent with the findings in this study and others^3,26^ that none of the teeth in the hadrosaurid dental battery are fused together, even in thin sections across entire batteries (Figs 1, 2). Each tooth retains a periodontium consisting of cementum, and alveolar bone. As the only extant toothed archosaurs, crocodilians provide support for the presence of a soft PDL that anchors the teeth in place in hadrosaurids as Sharpey’s fibers are observed in the cementum of both specimens (Fig. 3c,d,g,h). The PDL fibers in crocodilians anchor only into the cementum-coated portion of the tooth, which underlies the enamel-capped crown. Hadrosaurids differ not in the tissue types anchoring each tooth to the battery, but in the arrangements of these tissues relative to the crocodilian condition.

Despite the lack of an extant analogue for an entire battery, extant analogues can be found for individual hadrosaurid teeth. Unlike the teeth of modern crocodilians, the mammalian ever-growing incisor offers an almost exact comparison to the hadrosaurid tooth attachment tissue arrangements, which can then be used to explain battery-level changes and movement of teeth. The histological thin sections of the pika incisor show where we can confidently extrapolate the PDL connections to be in hadrosaurid teeth. In the pika, there is no PDL attachment to the enamel surface of the tooth, as the enamel-forming ameloblasts prevent the PDL fibers from anchoring to the enamel surface (Fig. 3o). Thin sections of a skeletonized rabbit show only a space where the PDL would have been in life, but still Sharpey’s fibers remain within the alveolar bone adjacent to the cementum of the incisor, and none are visible in the alveolar bone adjacent to the enamel (Fig. 5a,e). Thus, even in skeletonized specimens, it can be shown that the PDL anchored only to one side of the incisor in the rabbit. Similarly, this model explains why there are Sharpey’s fibers visible in the cementum of hadrosaurid teeth facing alveolar bone and other teeth, but none seen in the enamel-facing lingual plate of the jaw (Fig. 5b,f).

**Figure 8:**
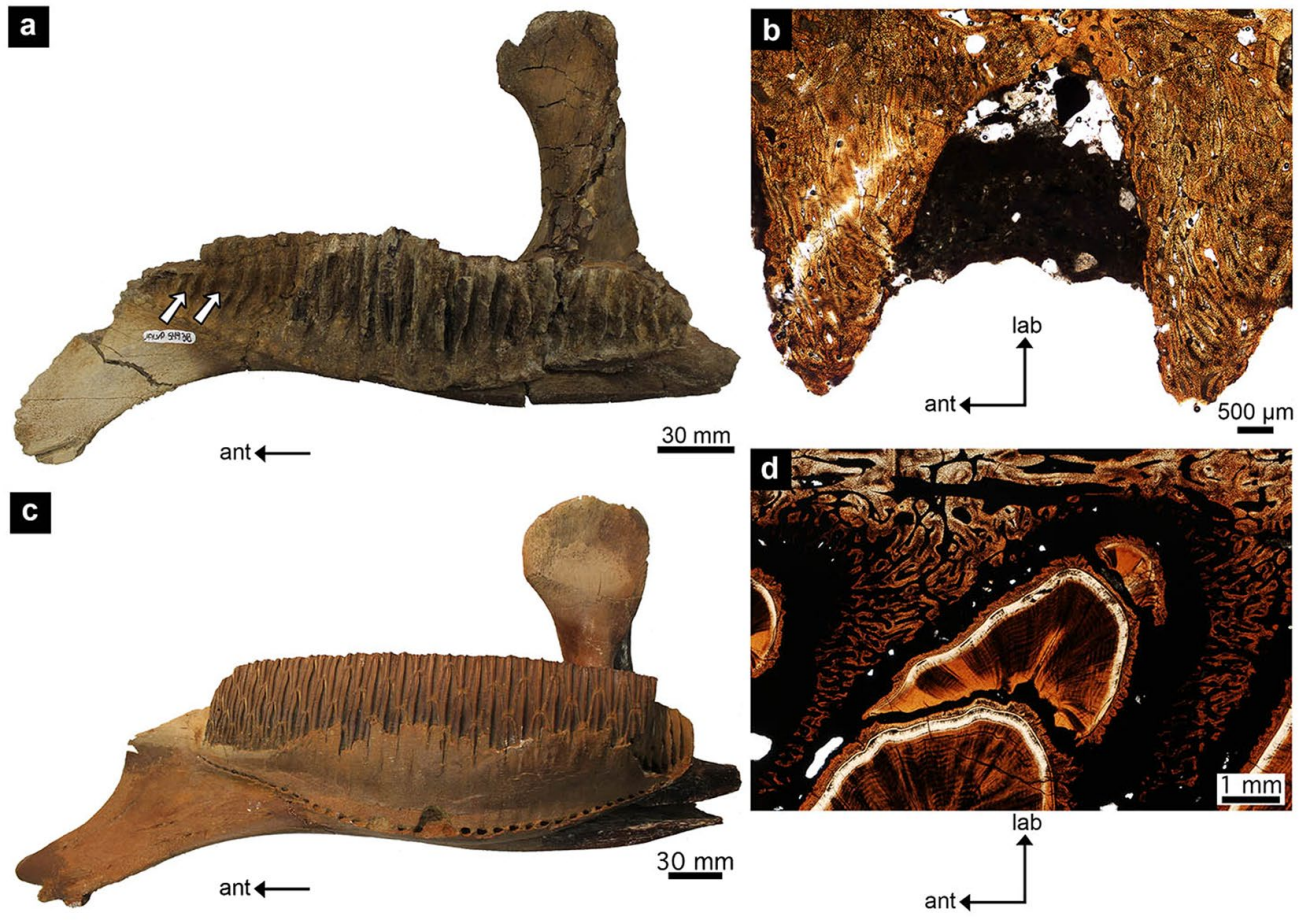
Hadrosaurid dentaries with and without teeth preserved. (**a**) UALVP 54938 without teeth; (**b**) UALVP 54812, histological thin section of a dental battery without teeth; (**c**) UALVP 11734 with teeth; (**d**) UALVP 56336, histological thin section of a dental battery with teeth. White arrows in (**a**) indicate a few of the alveolar septa. ant, anterior; db, dental battery; lab, labial.

The presence of a PDL connection of the teeth in hadrosaurids also explains the extreme variation in the state of preservation of hadrosaurid dental batteries observed in the field. Isolated hadrosaurid teeth are common, and so are edentulous jaws (Fig. 8a,b), which support the soft tissue model. But complete dental batteries are consistently found in the field (Fig. 8c,d), and these would seem to go against the soft tissue model. However, when complete batteries are recovered in the field, such as UALVP 56336, it is now clear that the infilling of the periodontal space with sediment during the fossilization process packs the teeth together, preventing the teeth from shedding or falling out of the jaw post-mortem.

In hadrosaurids, teeth within the occlusal surface are constantly being worn down, and the eruption rate must equal the attrition rate, as it does in the mammalian ever-growing incisor^24,27^. Due to their similarities, any eruption model for the mammalian ever-growing incisor is applicable for hadrosaurid teeth. Tooth eruption is a complex phenomenon, and while the exact mechanism of tooth eruption in the mammalian ever-growing incisor is still unknown^28,29^, there are two leading theories: (1) cellular proliferation at the base of the tooth^28,30^; and (2) tension from the periodontal ligament^28,29,31–33^.

For the cellular proliferation theory, pulpal tissue generating mitotic activity produces enough force to cause tooth eruption^28^. As hadrosaurid teeth do not preserve the pulpal tissues, this eruption theory is unable to be assessed for hadrosaurid dinosaurs. The second theory implicates the PDL as the eruptive force. As seen in the lagomorph thin sections, PDLs only attach to the cementum and thus only attach to the labial and lateral sides of the lagomorph – and rodent – incisors^24,25^. In the mammalian ever-growing incisor, these attachment tissues are constantly remodeled in order to support the continuous eruption and likely play a mechanical role in incisor eruption^28,29,31–33^. The collagen fibers of the PDL could potentially create tensional forces by forming at oblique angles and pull the tooth up as they contract^28,29^. For this to happen, there must be a continuous replacement of the PDL fibers^28,29^. Sharpey’s fibers of the hadrosaurid tooth are angled obliquely in coronal view, indicating the PDLs were embedded at an angle that could have supported eruption through the tension of collagen fiber contraction (Fig. 3c,d). Each individual hadrosaurid tooth may have its own eruptive forces; however, the PDL allowed the entire dental battery to erupt as a single unit through this unique tooth-tooth connection^3^ while also allowing some flexibility between the teeth to accommodate drift and tooth family addition.

The hadrosaurid histological sections showed multiple signs of anteroposterior tooth migration, a phenomenon this study recognizes in hadrosaurid dental batteries for the first time. Tooth migration is the process of tooth positions drifting within the jaw either during growth or as a way to maintain tooth contacts as the teeth are worn by an abrasive diet^34,35^. Tooth migration is rarely documented in reptiles, but is a common phenomenon in mammals and it is well known that the PDL accommodates this migration^35^. In mammals, when a force is applied to a tooth, causing it to drift, the tooth is surrounded by areas of alveolar bone remodeling. This is the process involved with orthodontic tooth movement^36^. The area of the tooth socket being remodeled in the direction of movement is the resorbing side^34,35^. On this side of the socket, osteoclasts resorb the alveolar bone and all mineralized material, detaching the PDL from its anchorage to the alveolar bone^34,35^. However, this resorption happens in asynchronous cycles where only a small proportion of the bone is being resorbed at one time, thus allowing a high percentage of PDLs of the tooth socket to maintain anchorage throughout migration^35^. Opposite to the resorbing side is the apposition side, where new bone is laid down by osteoblasts^34,35^. This new alveolar bone appears thin and delicate as it consists of woven bone trabeculae and large vascular canals^15^. The bone balance around the socket is always in equilibrium as the bone loss on the resorbing side is equal to the bone gain on the apposition side^35,37^. While the migration rate decreases after growth, it never ceases completely^34,35^.

The extensive remodeling of the alveolar bone, asymmetrical resorption of teeth, and the displacement of successive generations of teeth in the hadrosaurid dental battery provide clear evidence of tooth migration. Furthermore, the morphology of the alveolar septa and the displacement of successive tooth generations along the adult battery indicate that there were multiple directions and degrees of migration. There was a greater force moving the most anterior tooth families anteriorly than the more posterior tooth families. Moreover, the most posterior tooth families show evidence of posterior migration. In the perinatal dental battery all of the alveolar septa are angled anteriorly, suggesting that tooth migration begins early in ontogeny and that the posterior migration happens later in ontogeny.

The overall pattern of tooth migration demonstrates a high degree of dynamicity in the hadrosaurid dental battery. There is not one, but multiple directions of migration within the dental battery as the teeth react to forces applied unequally throughout the dental battery. Although the mechanisms behind tooth migration in the hadrosaurid dental battery require further investigation, opposition of forces during oral food processing may have had a strong influence. Recent work on hadrosaurid jaw mechanics suggests that they were palinal feeders with a medial rotation of the dentaries at the power stroke^38^. This method of oral food processing may account for the differing degree of tooth migration throughout the dentary dental battery.

Histological thin sections of lagomorph incisors contribute to a deeper understanding of hadrosaurid dental attachment tissues. While there is no extant analogue for the hadrosaurid dental battery, the lagomorph incisor is an excellent extant analogue for the individual hadrosaurid tooth. This study supports a more dynamic model for the dental battery^3^ by showing compelling evidence for soft tissue connections between all of the teeth within the hadrosaurid dental battery. Histological thin sections of full dental batteries, originally considered robust, solid structures, have revealed a high degree of three-dimensional tooth movement within the battery through ontogeny, a phenomenon that was only possible due to constant bone remodeling and the presence of PDLs. While the exact cause of tooth migration in the hadrosaurid dental battery is unknown, it could be the result of forces from multiple sources, such as accommodation of new tooth families and the opposition of forces during oral food processing. Given the similarities in the arrangement of tooth attachment tissues, future work on the mechanisms of tooth eruption in the mammalian ever-growing incisor can be used to further explain this phenomena in the complex dentitions of hadrosaurids.

## Methods

Histological thin sections were made of two hadrosaurid dentary dental batteries from the Upper Cretaceous of Dinosaur Provincial Park, Alberta, Canada. All cuts were made using the Buehler IsoMet 1000 slow-speed wafer blade. The perinatal dentary (UALVP 54419) and adult dentary (UALVP 56336) were cut transversely in two serial sections (Figs 1, 2). The perinatal dentary was embedded in a mixture of Castolite AC crystal clear polyester resin and degassed in a vacuum chamber. The adult dentary (UALVP 56336) was initially reinforced with Apoxie Sculpt self-hardening molding compound along any weak points. The hardened resin blocks were then cut before and after the embedding process. All specimens were mounted to frosted plexiglass slides using either CA-40 bonding compound (UALVP 54419) or Polymer solutions PSI 122 resin and PSI 124 hardener (UALVP 56336) and cut away from the embedded portion. They were then ground down using a Hillquist or a Crystal Master pro 12 lap wheel and further polished by hand using 600 and then 1000 grits of silicon carbide. These steps were repeated for the subsequent cuts of each dentary. The resulting thin sections were imaged using a Nikon DS-Fi-2 camera mounted to a Nikon AZ-100 microscope with NIS Elements BR imaging software (registered to D. C. Evans), or using a Nikon DS-Fi- 3 camera mounted to a Nikon Eclipse E600 POL microscope with NIS Elements D imaging software (registered to M. W. Caldwell).

A sagittal histological thin section of a modern rabbit (*Oryctolagus cuniculus;* UALVP 56918) lower incisor was made following the same protocol as the perinatal dentary. The incisor was first adhered to a basecoat of resin using CA-40 before being embedded to prevent the incisor from floating up during the degassing process in the vacuum chamber. For comparisons of tooth attachment in other archosaurs, previously made histological thin sections of a dentary of the extant crocodilian *Caiman sclerops* (made by Budney^39^) as well as an incisor of an extant pika (*Ochotona sp*.), from the histology slide collection of the Advanced Microscopy Facility, University of Alberta, were also examined. Images of these thin sections were taken using a Nikon DS-Fi- 3 camera mounted to a Nikon Eclipse E600 POL microscope with NIS Elements D imaging software.

Angles of the alveolar septa were measured from the longitudinal axis of the jaw to the apex-base segment of the alveolar septum using ImageJ^40^. The tip-base segment is a line extending from the tip of the septum to the middle of the septum’s base (Supplementary Information S1).

Due to the unique structure of the hadrosaurid dental battery, typical dental terminology becomes ambiguous. For this reason anterior and posterior are used rather than mesial and distal, and labial and lingual are used to represent the part of the tooth closest to the cheek side and tongue side, respectively. For the two serial sections made of each dental battery, the occlusal thin section refers to the top thin section, closest to the occlusal surface, and the basal thin section refers to the lower thin section, closer to the base of the jaw. The term alveolar septum refers to the alveolar bone extending between tooth families.

### Data availability

No datasets were generated or analysed during this study.

## Electronic supplementary material

Supplementary Information
