# Supplementary material for: Histological evidence for a dynamic dental battery in hadrosaurid dinosaurs
Source: Sci Rep. 2017 Nov 17;7:15787. doi: 10.1038/s41598-017-16056-3 (PMC5693932; doi:10.1038/s41598-017-16056-3)
Supplement: Supplementary file 1 — Supplementary Information [file 41598_2017_16056_MOESM1_ESM.pdf]

Histological evidence for a dynamic dental battery in hadrosaurid dinosaurs

Katherine Bramble, Aaron R. H. LeBlanc, Denis O. Lamoureux, Mateusz Wosik, Philip J. Currie

Supplementary Information

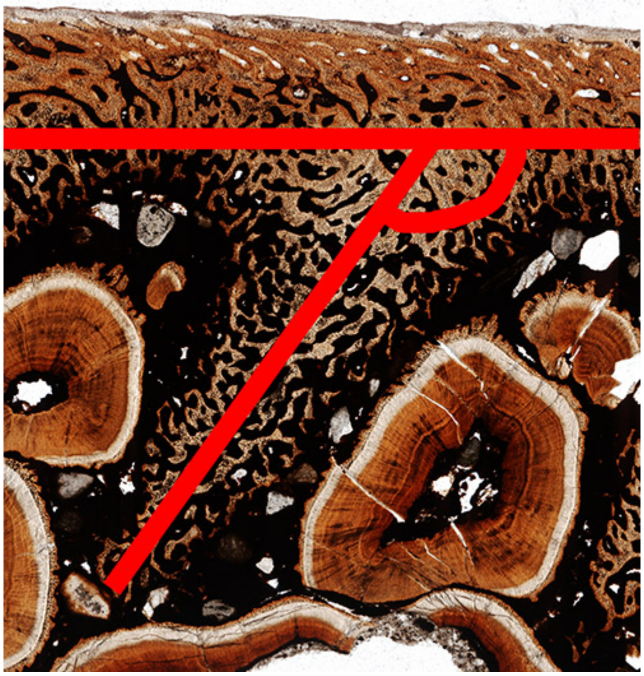

Figure S1. Visualization of the angles used for measuring the migration of alveolar septa.

Anterior is to the left of the image.
